# Fibronectin 1 Aggravates Colon Cancer Metastasis by Regulating RAP1B Protein Stability Through Akt/CREB Signalling Pathway

**DOI:** 10.1111/jcmm.70702

**Published:** 2025-07-10

**Authors:** Zhonghe Ji, Xinbao Li, Yadong Wang, Xinjing Zhang, Zhiran Yang, Yanbin Zhang, Junhui Yu, Chao Gao, Guojun Yan, Lijun Yan, Kai Zhang, Jinghan Pan, Songlin An

**Affiliations:** ^1^ Department of Peritoneal Oncology, Beijing Tsinghua Changgung Hospital, School of Clinical Medicine Tsinghua University Beijing China; ^2^ Department of Peritoneal Cancer Surgery, Beijing Shijitan Hospital Capital Medical University Beijing China; ^3^ Scientific Research Department GeneX Health Co. Ltd. Beijing China

**Keywords:** Akt/CREB signalling, colon cancer, fibronectin 1, metastasis

## Abstract

Metastasis is one of the important factors leading to poor prognosis in patients with colon cancer. However, the molecular mechanism contributing to this cellular behaviour remains largely unknown. Here, RNA‐seq analysis suggested that Fibronectin 1 (FN1) was significantly increased in metastatic colon cancer, which was associated with poor prognosis. FN1 enhanced colon cancer cell migration, invasion, and epithelial to mesenchymal transition (EMT) in vitro and promoted liver and lung metastasis of colon cancer in nude mice through RAP1B. Mechanistically, FN1 could interact with RAP1B by suppressing the interaction between RAP1B and the E3 ligase PARK2, relieving RAP1B ubiquitination modification and improving RAP1B protein stability. Furthermore, FN1‐RAP1B activated Akt signalling pathway, leading to the phosphorylation and activation of CREB. Interestingly, CREB was found directly bound to the FN1 and TIMP1 promoter, respectively and increased FN1 and TIMP1 transcription, thereby establishing a positive regulatory feedback loop. Overall, our results elucidated that FN1 promotes colon cancer migration, invasion, and metastasis. FN1 may be used as the potential therapeutic target for colon cancer metastasis.

## 1 Introduction

Colon cancer is one of the most common malignancies worldwide, of which metastasis is the main factor of poor prognosis of colon cancer patients [1, 2, 3, 4]. Despite of radiotherapy and chemotherapy applied in the treatment of metastatic colon cancer, the survival was still unsatisfied [5, 6, 7]. Metastatic colon cancer commonly occurs in the liver, lungs, bones and so on. Since the metastatic occurrence of primary colon cancer is directly related to the survival and affects about 90% of all colon cancer deaths [6, 8, 9], it's of great importance to deeply investigate the molecular mechanism of colon cancer metastasis and discover effective targets for metastatic colon cancer treatment.

Extracellular matrix protein fibronectin (FN1) has been implicated in cell adhesion and migration processes including embryogenesis, wound healing, blood coagulation, host defence, and metastasis [10, 11]. Recent studies revealed that FN1 has been reported increased in a variety of tumours. Integrated analysis of circulating and tissue proteomes reveals that FN1 is a potential biomarker in papillary thyroid cancer [12]. High FN1 expression is associated with poor survival in oesophageal squamous cell carcinoma [13]. FN1 was reported to mediate the activation of aspartate metabolism and promote the progression of triple‐negative and luminal a breast cancer [14]. It was found that FN1 was also important in colon cancer proliferation and progression [15, 16]. Yet, the underlying mechanism of FN1 in colon cancer metastasis was largely unknown.

RAP1B is a member of RAS oncogene family, which regulates multiple cellular processes including cell adhesion and growth and differentiation. Recent studies have shown that RAP1B was involved in multiple tumours progression [17]. RAP1B was reported to be the target of miR‐206 in regulating thyroid cancer proliferation and invasion [18]. It was found that RAP1B was associated with poor prognosis and promoted an aggressive phenotype in gastric cancer [19]. RAP1B was also reported to be vital for the tumorigenesis and chemoresistance of laryngeal cancer [20]. An earlier study showed that RAP1B was involved in intestinal inflammation [21]. However, there was little research about the role of RAP1B in colon cancer progression and metastasis.

In this study, we investigated the role of fibronectin 1 (FN1) in facilitating colon cancer metastasis. FN1 could regulate colon cancer migration, invasion, EMT, and metastasis by regulating RAP1B. Mechanistically, FN1 interacted with RAP1B and suppressed the interaction between RAP1B and PARK2, thereby promoting RAP1B protein stability and relieving the ubiquitination modification.

## 2 Methods

### 2.1 Human Colon Cancer Samples

All human tumour samples of colon cancer were obtained from Beijing Shijitan Hospital affiliated Capital Medical University (sjtky11‐1x‐2021(12)). Patients before the surgery signed the written informed consent for the clinical and research studies. The study was approved by the ethical committee.

### 2.2 RNA Sequencing and High‐Throughput Sequencing Data Collection

The samples were ground in liquid nitrogen, followed by the extraction of total RNA using Trizol reagent (Invitrogen, USA). The purity and quality of the RNAs were assessed using the Thermo NanoDrop 2000 spectrophotometer (Wilmington, USA) and agarose gel electrophoresis. Subsequently, mRNA was enriched with oligo(dT) beads and randomly fragmented in a fragment buffer. The first cDNA was synthesised using random hexamers and reverse transcriptase. Following this, dNTPs, RNase H, and *Escherichia coli* polymerase I were added to the second‐strand synthesis buffer (Illumina, USA) to generate the second strand through nick translation. A cDNA library was then established through purification, terminal repair, A‐tailing, ligation of sequencing adapters, size selection, and PCR enrichment among other steps. The established cDNA library underwent sequencing using the Illumina HiSeq X Ten RNA‐seq platform (Illumina, USA), resulting in raw data that was converted into sequence readings. This raw data was recorded in a FASTQ file containing sequence information (reads) as well as corresponding sequencing quality details. Finally, the raw reads were filtered to remove those containing adapters or of low quality before being mapped to the human reference genome (hg38) utilising hisat2 software for further analysis.

We have also acquired high‐throughput sequenced gene expression data and clinical information from public databases for patients with colon adenocarcinoma (COAD) to conduct gene expression validation and survival analysis for patients, to explore gene expression patterns and their correlation with patient survival. The TCGA‐COAD dataset consists of 339 tumour tissues and 43 normal tissues. Among these, there are 280 patients diagnosed with stage M0 and 59 patients diagnosed with stage M1 according to the stage of remote metastasis of case parameters. We downloaded the GSE17536 COAD dataset from the NCBI GEO database (https://www.ncbi.nlm.nih.gov/geo/) [22]. The GSE17536 dataset includes 177 patients and was sequenced using the GPL570 [HG‐U133_Plus_2] Affymetrix Human Genome U133 Plus 2.0 Array chip.

### 2.3 Gene Expression Differences Analysis

The limma R package was utilised to analyse the differences in gene expression among primary (P) COAD, metastatic (M) COAD, and normal tissues (N) [23]. A threshold of false discovery rate (FDR) < 0.05 and relative expression value (|log2 fold change (FC)|) ≥ 1.5 was applied to identify differentially expressed genes (DEGs). The intersection analysis of DEGs between PvsN, MvsN, and MvsP was carried out to obtain the common DEGs. The findings from the analysis of genetic differences were presented using the ggplot2 and heatmap R packages, in the form of heat maps and volcano maps [24].

### 2.4 Survival Analysis

Survival analysis was conducted using gene expression data and patient clinical information from the TCGA and GSE17536 datasets. The gene expression profile was normalised through log2 transformation, and the median gene expression level was used as the cut‐off value to divide the samples into high‐ and low‐level groups. The Kaplan–Meier method was employed to assess and plot the survival curve of genes, with “survival” and “survminer” software packages in R utilised for log‐rank test. A statistically significant difference was defined as *p* < 0.05 for the cut‐off criterion. Additionally, the risk ratio (HR) with 95% confidence intervals (CI) was calculated. Finally, the correlation between gene expression and patients' overall survival (OS), disease‐specific survival (DSS), disease‐free survival (DFS), and recurrence‐free survival (RFS) was determined.

### 2.5 Correlation and KEGG Analysis

By applying |Pearson *R*| > 0.5 and *p* < 0.01 as thresholds, we conducted Pearson correlation analysis to identify the co‐expressed genes associated with FN1. Enrichment analysis of biological pathways was performed using the R language package ClusterProfile, based on Kyoto Encyclopedia of Genes and Genomes (KEGG), with a significance level set at *p* < 0.05 [25]. The results were statistically significant and visualised using the ggplot2 package in R, presenting the top enriched KEGG pathways in a bar plot.

### 2.6 Gene‐Set Enrichment Analysis of RAP1B

The gene expression profile was normalised through log2 transformation, with the median gene expression level of RAP1B serving as the cutoff value to divide patient samples into high‐ and low‐ level groups. The entire gene set of KEGG (c2.cp.kegg.7.5.1. symbols) from *Homo sapiens* was obtained from the GSEA database (http://www.gsea‐msigdb.org/gsea/). Subsequently, the normalised expression data of RNAseq and TCGA were uploaded to GSEA 4.3.3 software for analysis. Using signal‐to‐noise ratio measurements in GSEA, a total of 20,530 genes were sequenced based on their correlation with RAP1B. Gene sets containing less than 5 or more than 2000 genes were excluded from the analysis. To evaluate the enrichment degree and statistical significance, a normalised enrichment score (NES) > 1 and false discovery rate (FDR) < 0.25 were utilised. As a result, the enrichment pathways of RAP1B were identified.

### 2.7 Culture of Patient‐Derived Tumour‐Like Cell Clusters (PTCs)

The patient‐derived tumour‐like cell clusters (PTCs) were from colon cancer tissues. The collected tissue samples were cultured in the ice‐cold PBS with 10 mM HEPES and 100 U/mL penicillin–streptomycin (Thermo Fisher Scientific). Tissues were washed with PBS and the necrotic tissues were dissected off as much as possible. Tissues were cut into small pieces and digested in 1 mM PBS/EDTA containing 200 U/mL collagenase I, II, and IV (Thermo Fisher Scientific) at 37°C for 1 h. The dissociated cells were collected using the 40 μm filters. Cells (10^5^ cells/cm^2^) after centrifugation were seeded and cultured in a low‐attachment‐surface dish at 37°C with 5% CO_2_. The PTC growth medium was changed every 2 or 3 days.

### 2.8 Cell Culture

Human colon cancer HCT116 cells were cultured in McCoy's 5A medium. Human colon cancer LOVO cells were maintained in Ham's F‐12 K medium. All the culture medium was supplemented with 10% foetal bovine serum (FBS), 100 U/mL of penicillin and 100 μg/mL of streptomycin.

### 2.9 Plasmids and Short Hairpin RNA (shRNA)

The FN1, RAP1B, and CREB lentiviral shRNA plasmids were supplied by GenePharma (Shanghai, China). The sequence information was listed in Table 1. Cell transfection was performed using Lipofectamine 2000 (Invitrogen).

**TABLE 1:** Sequences of shRNAs.

shRNAs		Sequences
shNC	Oligo 1	5′‐ACCTCGGATAGTGCGGGTATTGTGATTCAAGAGATCACAATACCCGCACTATCCTT‐3′
	Oligo 2	5′‐CAAAAAGGATAGTGCGGGTATTGTGATCTCTTGAATCACAATACCCGCACTATCCG‐3′
shFN1 1#	Oligo 1	5′‐ACCTCGGCTGGATGATGGTAGATTGTTCAAGAGACAATCTACCATCATCCAGCCTT‐3′
	Oligo 2	5′‐CAAAAAGGCTGGATGATGGTAGATTGTCTCTTGAACAATCTACCATCATCCAGCCG‐3′
shFN1 2#	Oligo 1	5′‐ACCTCGGAGCTCTATTCCACCTTACATCAAGAGTGTAAGGTGGAATAGAGCTCCTT‐3′
	Oligo 2	5′‐CAAAAAGGAGCTCTATTCCACCTTACACTCTTGATGTAAGGTGGAATAGAGCTCCG‐3′
shRAP1B 1#	Oligo 1	5′‐ACCTCGTCCACATTTAACGATTTACATCAAGAGTGTAAATCGTTAAATGTGGACTT‐3′
	Oligo 2	5′‐CAAAAAGTCCACATTTAACGATTTACACTCTTGATGTAAATCGTTAAATGTGGACG‐3′
shRAP1B 2#	Oligo 1	5′‐ACCTCGCATTAGTTTATTCCATCACATCAAGAGTGTGATGGAATAAACTAATGCTT‐3′
	Oligo 2	5′‐CAAAAAGCATTAGTTTATTCCATCACACTCTTGATGTGATGGAATAAACTAATGCG‐3′
shCREB	Oligo 1	5′‐ACCTCGCAATACAGCTGGCTAACAATTCAAGAGATTGTTAGCCAGCTGTATTGCTT‐3′
	Oligo 2	5′‐CAAAAAGCAATACAGCTGGCTAACAATCTCTTGAATTGTTAGCCAGCTGTATTGCG‐3′

### 2.10 Quantitative Real‐Time PCR (qPCR)

Total RNA was extracted using Trizol (Invitrogen, Carlsbad, CA). 1 μg total RNA was reverse transcribed into cDNA using the RT kit (Takara, Shiga, Japan). PCR analysis was performed using a 7500 Realtime PCR System (Applied Biosystems, Carlsbad, CA, USA). The relative RNA expression was normalised to GAPDH, which was calculated by the 2^−ΔΔCt^ method. The primers are shown in Table 2.

**TABLE 2:** Primers for qPCR.

Genes		Sequences (5′ → 3′)
FN1	Forward	AGGAAGCCGAGGTTTTAACTG
	Reverse	AGGACGCTCATAAGTGTCACC
RAP1B	Forward	AGCAAGACAATGGAACAACTGT
	Reverse	TGCCGCACTAGGTCATAAAAG
GAPDH	Forward	GGAGCGAGATCCCTCCAAAAT
	Reverse	GGCTGTTGTCATACTTCTCATGG

### 2.11 Western Blot

Cells were lysed in RIPA lysis buffer with a proteinase inhibitor. Protein concentrations were quantified with the BCA Protein Assay Kit (Beyotime Biotechnology, China). Proteins (20 μg) were separated by sodium dodecyl sulfate polyacrylamide gel electrophoresis (SDS‐PAGE) and transferred onto polyvinylidene fluoride (PVDF) membranes. Membranes were blocked in TBST containing 5% non‐fat milk at room temperature (RT) and then incubated with the indicated primary antibodies at 4°C overnight. Membranes after washing were incubated with secondary antibodies for 2 h at RT. After washing again with TBST three times, the bands were detected using an enhanced chemiluminescence (ECL) kit (Beyotime Biotechnology, Shanghai, China).

### 2.12 Co‐Immunoprecipitation

Cell lysates were centrifuged and the supernatant was obtained. The supernatant was incubated with indicated antibodies or IgG controls at 4°C overnight. The next day, the mixture was incubated with the protein A agarose (Sigma Aldrich, P3476) for another 2 h at 4°C. The mixture (bead‐antibody‐protein complex) was washed with ice‐cold PBS buffer and harvested for western blot analysis.

### 2.13 Wound Healing Assay

At a density of 5 × 10^5^/mL, the colon cancer cells were seeded into 6‐well plates. The formed confluent monolayer was scratched with a 200 μL sterile pipette tip. Then, the cells were cultured in fresh medium supplemented with 2% FBS. The status of cell migration at 0 h and 24 h was photographed using the Olympus microscope (20×, Tokyo, Japan) equipped with a digital camera, and the images were analysed by Image J. Cell migration was represented by the wound width compared to that at 0 h.

### 2.14 Luciferase Assay

The FN1/TIMP1 wild‐type or mutant promoter was cloned into the luciferase reporter vector. Cells were grown in 24‐well plates and co‐transfected with the corresponding reporter plasmids in each group. The Dual‐Luciferase Reporter Assay (Promega) system was used to detect the luciferase activity.

### 2.15 Transwell Assays

Cell migration and invasion assays were performed by transwell insert chambers with 8‐μm pore membranes (Corning, NY, USA). Approximately 0.5 × 10^5^ cells were seeded in the upper chamber with serum‐free medium containing 0.2% bovine serum albumin (BSA). The culture medium with 10% FBS were added in the lower chamber. After 24 h, cells were scraped off from the upper surface of the upper chamber. Then the chamber surface was washed with PBS, fixed with formalin, and stained with crystal violet staining solution. The migration cells were photographed under a microscope. For invasion assay, the chambers with inserts were coated with Matrigel (100 μg/mL, 15 μL/well, BD, 356230), the following steps were as described above.

### 2.16 In Vivo Colon Cancer Metastasis Model

Colon cancer metastasis mice model were established by injecting 1 × 10^6^ colon cancer cells (HCT116 or LOVO) via the tail vein of NSG mice. The mice were randomly divided into four groups, in which the colon cancer cells were infected with control and shNC lentivirus, control and shRAP1B lentivirus, FN1 and shNC lentivirus, and FN1 and shRAP1B lentivirus for 48 h, respectively. For imaging assay, all cells were infected with lentivirus labelled with GFP‐fluorescence for 48 h. The distribution of colon cancer cells was traced and analysed using an in vivo imaging system (Kodak, Effingham, IL, USA). The study was approved by the Ethics Committee of Beijing Shijitan Hospital affiliated Capital Medical University.

### 2.17 Statistical Analysis

All data are presented as the mean ± SD. Two‐tailed Student's *t* test was performed for the statistical comparison. Overall survivals were analysed using the Kaplan–Meier method. *p* < 0.05 was statistical significance.

## 3 Results

### 3.1 FN1 Is Elevated in Primary and Metastatic Colon Cancer and Correlated With Poor Prognosis

**FIGURE 1:**
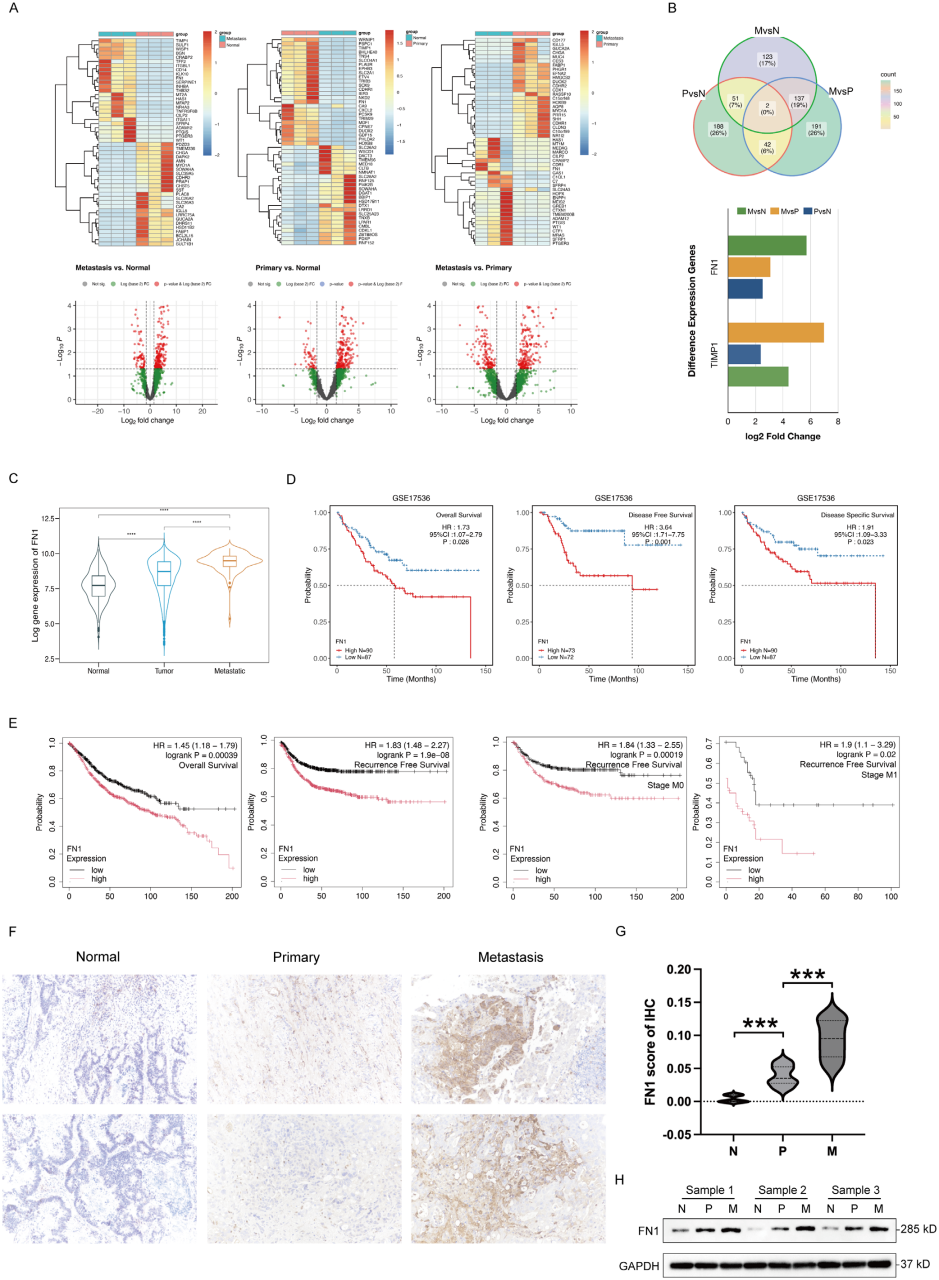
Identification of metastatic and prognostic‐related DEGs in colon cancer. (A) Heat maps and volcano maps displayed the DEGs between primary cancer, metastatic cancer, and normal tissue in the RNAseq data. The heat map depicted the expression of the first 50 DEGs. The *x* and *y* axes of the volcano map represented log2‐fold change and −log10 (*p* value) for each gene, respectively, with truncation of significance (*p* value = 0.05). The red, green, and grey dots denoted significant, nonsignificant, and non‐differential genes. (B) Common Venn diagrams showed the intersection of DEGs. (C) Expression of FN1 in TCGA colon cancer dataset. (D) Survival analysis of FN1 in GSE17536 dataset. (E) Survival analysis of FN1 in colon cancer‐TCGA. (F, G) Immunohistochemistry analysis of FN1 in colon cancer tumour tissues. Scale bars, 50 μm. *** *p* < 0.001. (H) Western blot analysis of FN1 in three pairs of metastatic, primary colon cancer tumour tissues and normal tissues.

To explore the specific genes associated with the peritoneal metastasis of colon cancer, firstly we performed transcriptome sequencing to analyse gene expression differences among 3 paired of metastatic, primary colon cancer tissues and their adjacent normal tissues. The RNA‐seq results revealed that 226 genes were significantly up‐regulated and 57 genes were significantly down‐regulated in metastasis tissues compared with normal tissues. Furthermore, compared with normal tissues, 232 genes were significantly up‐regulated and 81 genes were significantly down‐regulated in primary tumour tissues. In comparison with primary tumour, 222 genes were significantly up‐regulated and 150 genes were significantly down‐regulated in metastatic tumour tissues (Figure 1A). Intersection analysis of these DEGs indicated that the expression of two DEGs (TIMP1, FN1) differed significantly among the three groups, showing an upward trend in expression levels with tumour progression (Figure 1B). The expression of FN1 was validated highly expressed most in metastatic tumour tissues and much more highly expressed in primary tumour tissues compared to normal tissues from the TCGA dataset (Figure 1C).

Subsequently, we investigated the relationship between FN1 expression and overall survival (OS). The analysis in the GSE17536 dataset demonstrated that significant differences in OS (HR = 1.73, *p* = 0.026), DFS (HR = 3.64, *p* = 0.001), and DSS (HR = 1.91, *p* = 0.023) between low and high FN1 expression groups (Figure 1D). Similarly, high FN1 expression in the TCGA dataset was also significantly associated with poor OS of patients (HR = 1.45, *p* < 0.001), RFS (HR = 1.83, *p* = 1.9e‐08), as well as M0 (HR = 1.84, *p* < 0.001) and M1 (HR = 1.9, *p* = 0.02) stage subgroups, where patients with low FN1 expression exhibited notably better survival outcomes compared to those with high expression levels (Figure 1E). These results suggest that FN1 is implicated in tumour metastasis and serves as a prognostic factor for patient survival outcome.

Furthermore, we validated the expression of FN1 in 3 paired of colon cancer tissues. The immunohistochemical result showed that FN1 was mainly located expressed in primary and metastasis colon cancer tissues compared to adjacent normal tissues (Figure 1F,G), which was consistent with the western blot assay (Figure 1H). Overall, these results suggest that highly expressed FN1 is closely related to the metastasis of colon cancer and correlates with poor prognosis in colon cancer.

### 3.2 FN1 Promotes Colon Cancer Cell Migration, Invasion, EMT In Vitro

**FIGURE 2:**
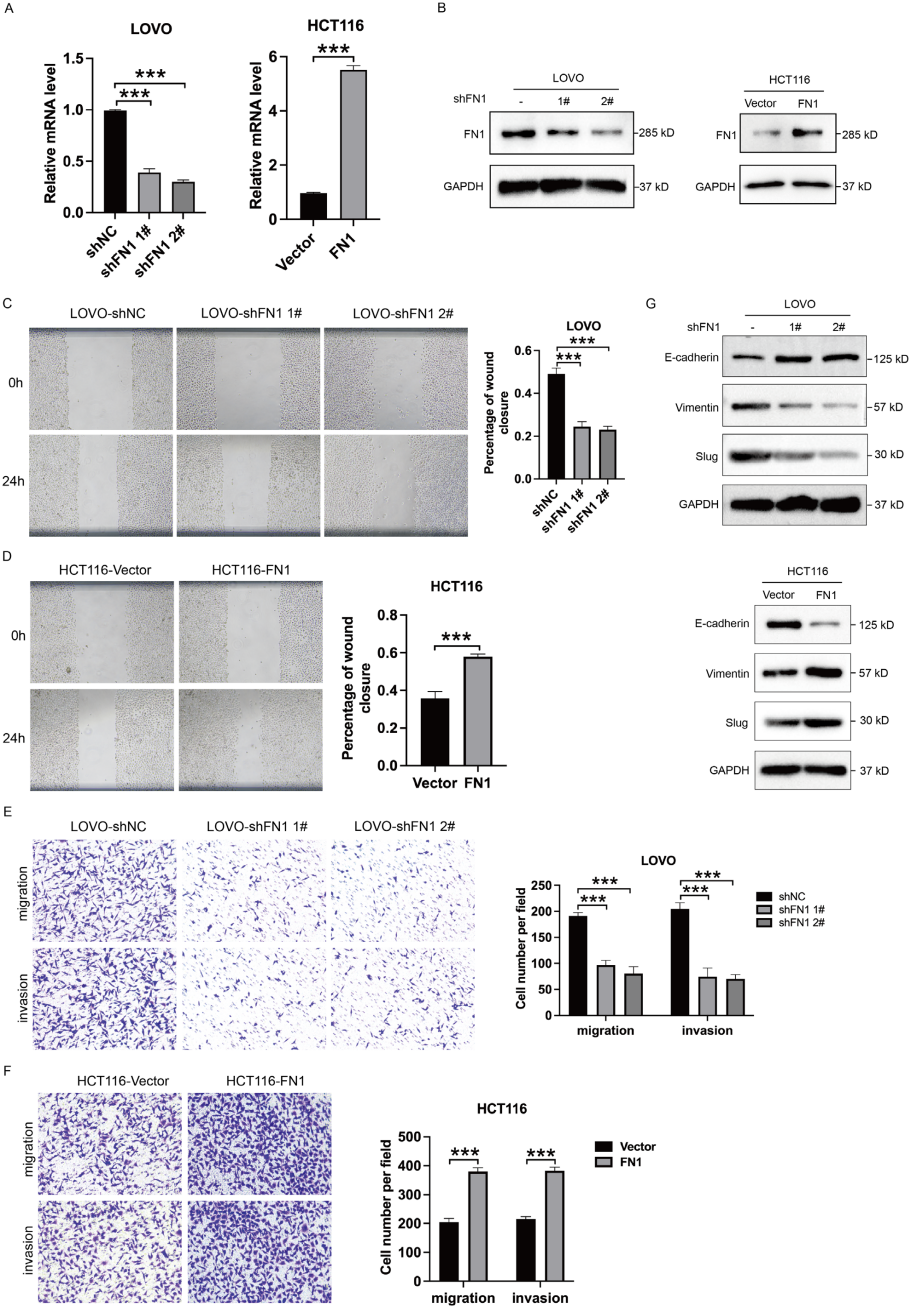
FN1 promotes colon cancer cell migration, invasion, EMT in vitro. (A, B) qPCR and western blot analysis showing the expression of FN1 in LOVO‐shNC/LOVO‐shFN1#1/LOVO‐shFN1#2 and HCT116‐vector/HCT116‐FN1 cell lines. Total GAPDH was used as the loading control. (C, D) Wound healing analysis of LOVO‐shNC/LOVO‐shFN1#1/LOVO‐shFN1#2 and HCT116‐vector/HCT116‐FN1 at 0 and 24 h. Representative images (left panel) and quantification (right panel) are shown as indicated. (E, F) Transwell and transwell‐matrigel assays were performed to assess migration and invasion ability of FN1‐knockdown and FN1‐overexpression cell lines. Representative images (left panel) and quantification (right panel) are shown as indicated. (G) Western blot analysis of EMT‐related proteins for LOVO‐shNC/LOVO‐shFN1#1/LOVO‐shFN1#2 and HCT116‐vector/HCT116‐FN1. ***p < 0.001.

Next, we performed loss‐of‐function and gain‐of‐function experiments to further investigate the functional effects of FN1 in colon cancer cells. We established stable FN1‐knockdown LOVO cell line using two short hairpin RNAs‐mediated gene silencing (shFN1 1# and shFN1 2#) and FN1‐overexpresssion HCT116 cell line by transfecting FN1‐overexpressing‐plasmid (Figure 2A,B). Wound healing experiments showed that FN1‐knockdown cells exhibited poor healing capability while FN1‐overexpression cells had better healing ability compared to control (Figure 2C,D). At the same time, the Transwell assay results showed that FN1‐knockdown cells exhibited slower cell migratory abilities whereas FN1‐overexpression cells had faster cell migratory abilities (Figure 2E). Transwell‐matrigel assay suggested that FN1 downregulation reduced cell invasion while FN1 overexpression promoted cell invasion (Figure 2F). Next, we examined the expression of epithelial‐mesenchymal transition (EMT) related markers. As expected, the silence of FN1 inhibited mesenchymal‐related markers Vimentin and Slug expressions and increased epithelial‐related marker E‐cadherin expression, overexpression had the opposite effect (Figure 2G). These data indicate that FN1 is a positive regulator of migration, invasion and EMT of colon cancer in vitro.

### 3.3 FN1 Interacts With RAP1B to Improve the Stability of the RAP1B Protein

**FIGURE 3:**
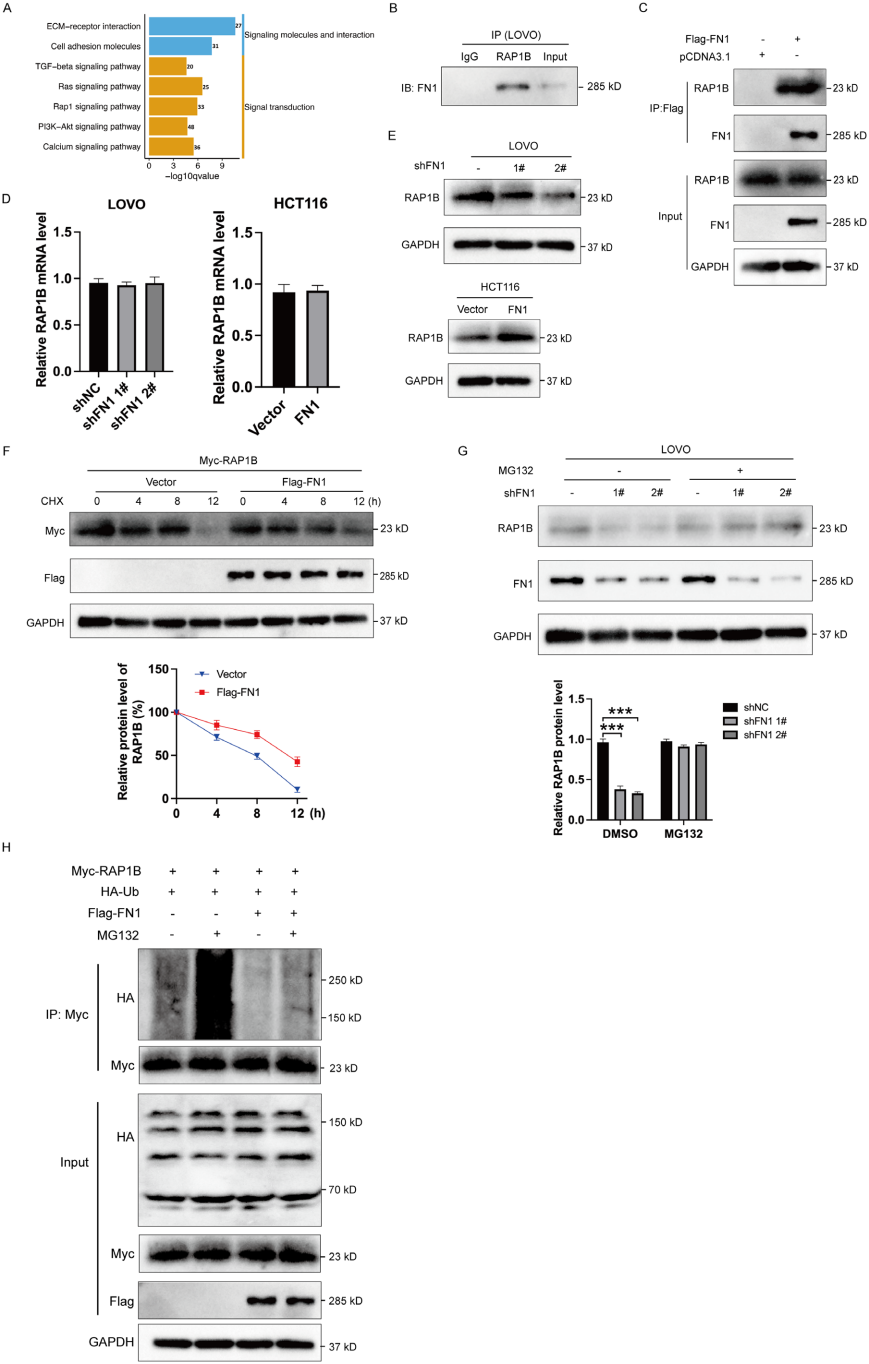
FN1 interacts with RAP1B to improve the stability of the RAP1B protein. (A) KEGG enrichment pathway of FN1 shown as the bar diagram. Blue indicates signal molecules and interaction subsets, and orange indicates signal transduction subsets. The number indicates the number of genes enriched. The *X*‐axis represented the significant *q* value of −log10 enrichment. (B) Immunoprecipitation of the FN1 protein using the anti‐RAP1B antibody in LOVO cells. IgG was used as the negative control. (C) Immunoprecipitation of the RAP1B protein by an anti‐Flag antibody in HEK293 cells transfected with pcDNA3.1‐FLAG‐FN1. pcDNA3.1‐vector was used as a negative control. (D) qPCR and western blot analysis of RAP1B expression at mRNA and protein levels in LOVO and HCT116 cells. (E) Western blot analysis of the RAP1B protein with the treatment of 200 μg/mL CHX at the indicated time, which transfected with Flag‐FN1 and Myc‐RAP1B in HEK293T cells. (F) Western blot analysis of RAP1B protein level in HEK293T transfected with Myc‐RAP1B and Flag‐FN1 plasmids with the treatment of CHX (200 μg/mL) at the indicated time points. (G) Western blot analysis of RAP1B in LOVO cells transfected with or without shFN1 and/or the MG132 treatment (20 μM, 4 h). *** *p* < 0.001. (H) Ubiquitination assay of RAP1B was performed in 293 T cells transfected with Myc‐RAP1B and HA‐Ub, with or without Flag‐FN1 and MG132.

The enrichment analysis revealed that FN1 is predominantly involved in the Ras signalling pathway (hsa04014, *p* = 2.81E‐07), Rap1 signalling pathway (hsa04015, *p* = 1.15E‐06), PI3K‐Akt signalling pathway (hsa04151, *p* = 1.93E‐05), as well as other pathways (Figure 3A). These results indicate a strong association between FN1 and Ras and RAP1 signals in colon cancer tumour progression. To elucidate the possible molecular regulatory mechanism underlying FN1 in colon cancer, we predicted that RAP1B was the potential interaction factor of FN1 via biogrid (https://thebiogrid.org/). The protein–protein interaction between FN1 and RAP1B was firstly validated by endogenous immunoprecipitation (IP) (Figure 3B). We found that there was a binding between FN1 and RAP1B (Figure 3B). Next, we used the anti‐Flag antibody to perform the co‐IP experiment by transiently transfecting HEK293T cells with Flag‐tagged FN1‐overexpression plasmids. The results demonstrated that FN1 could interacted with RAP1B in these cells (Figure 3C). Next, we assessed the effect of FN1 on RAP1B mRNA and protein expression. Western blot showed that the protein level of RAP1B was decreased in FN1‐knockdown cells and increased in FN1‐overexpressing cells whereas the mRNA level was not changed (Figure 3D,E). We wondered whether FN1 modulated RAP1B protein stability. Firstly, we did protein stability test in cells transfected with Flag‐tagged FN1 and Myc‐tagged RAP1B and treated with the protein synthesis inhibitor Cycloheximide (CHX) at different times, which could block protein synthesis. It was found that FN1 overexpression could attenuate the protein degradation of RAP1B in contrast to the control (Figure 3F). To further confirm the regulation of FN1 on RAP1B protein stability, we treated cells with the proteasome inhibitor MG132. Results showed that with MG132 treatment, the protein degradation of RAP1B slowed down in FN1‐knockdown cells than control (Figure 3G). We also performed the ubiquitination assay in HEK293T transfected with Myc‐RAP1B and HA‐Ub with or without Flag‐FN1 and/or MG132. The ubiquitination of RAP1B was higher in the absence of FN1 (Figure 3H, lane 2). The presence of FN1 could significantly decreased RAP1B‐ubiquitination (Figure 3H, lane 3–4). These findings suggest that FN1 could bind to and positively regulate RAP1B expression by maintaining RAP1B protein stability via the proteasome system.

### 3.4 FN1 Inhibits the Interaction Between RAP1B and PARK2 to Prevent RAP1B Ubiquitination

**FIGURE 4:**
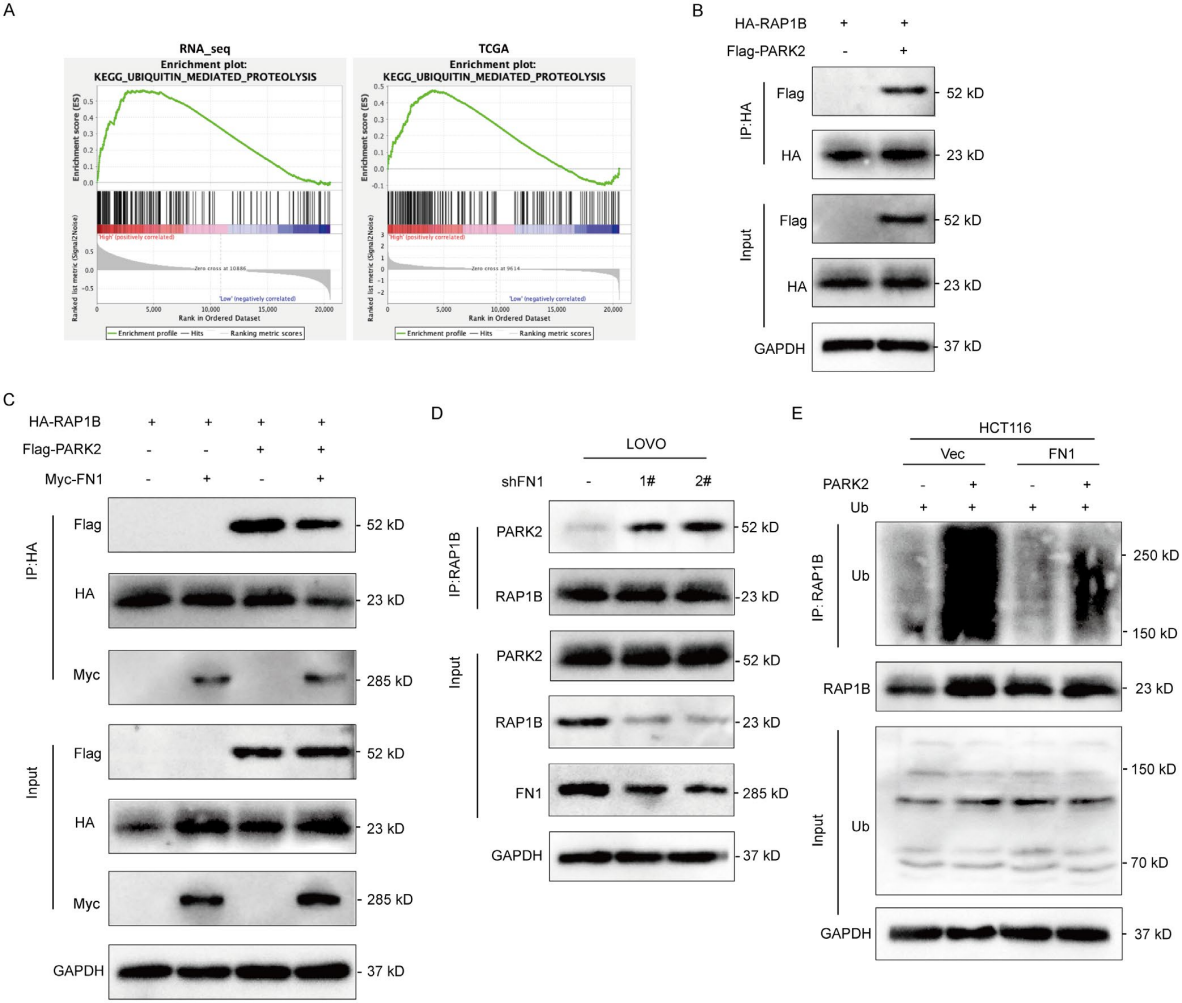
FN1 inhibits the interaction between RAP1B and PARK2 to prevent RAP1B ubiquitination. (A) GSEA analysis of RAP1B. Significantly enriched KEGG pathways in the high‐level group of RNA‐seq data and TCGA dataset, respectively. (B) Co‐IP experiment of cells transfected with Flag‐PARK2 and HA‐RAP1B plasmids. (C) Western blot detection of Flag, HA, Myc tagged proteins after immunoprecipitation in cells co‐transfected with HA‐RAP1B, Flag‐PARK2, and Myc‐FN1. (D) Co‐IP of PARK2 in LOVO‐shNC/LOVO‐shFN1#1/LOVO‐shFN1#2 using anti‐RAP1B antibody. (E) The ubiquitination assay of cells transfected with Flag‐PARK2 in HCT116‐vector/HCT116‐FN1 cells.

Next, we wondered whether FN1 mediated RAP1B ubiquitination. Patients with RNAseq and TCGA data were divided into high and low expression groups. The GSEA method was employed to perform enrichment analysis of KEGG pathways in both groups. GSEA analysis of RAP1B related genes revealed that ubiquitination pathway was highly enriched (NES > 1, FDR < 0.25, Figure 4A and Table S1), suggesting a strong likelihood for RAP1B to be involved in the ubiquitination pathway. The E3 ubiquitin protein ligase PARK2 was found to be the potential interacting molecule of RAP1B through biogrid (https://thebiogrid.org/). We assumed that there was an interaction between RAP1B and PARK2 and performed the IP experiment using an anti‐HA antibody. The immunoblotting analysis showed that HA‐RAP1B could pull down Flag‐PARK2 (Figure 4B). Furthermore, we co‐transfected 293 T cells with HA‐RAP1B, Flag‐PARK2, and/or with Myc‐FN1. The interaction between RAP1B and PARK2 was found to be decreased when co‐transfection with Myc‐FN1 (Figure 4C). On the other hand, we did co‐IP assay in FN1‐knockdown cells incubated with anti‐RAP1B antibody. The results showed that the binding between RAP1B and PARK2 was increased when FN1 was knockdown (Figure 4D). These data suggest that FN1 could prevent RAP1B binding to PARK2. Moreover, we performed the ubiquitination experiment in HCT116 cells transfected with HA‐Ub and/or exogenous PARK2 in the presence or the absence of Flag‐FN1. Results showed that with PARK2 overexpression increased the level of ubiquitin bound by RAP1B, and the ubiquitination level of RAP1B was weakened in HCT116 cells in the presence of FN1 (Figure 4E). These data suggest that RAP1B and PARK2 interact with each other. The presence of FN1 prevents RAP1B from being ubiquitinated induced by PARK2.

### 3.5 FN1 Promotes Cell Migration, Invasion and EMT via RAP1B

**FIGURE 5:**
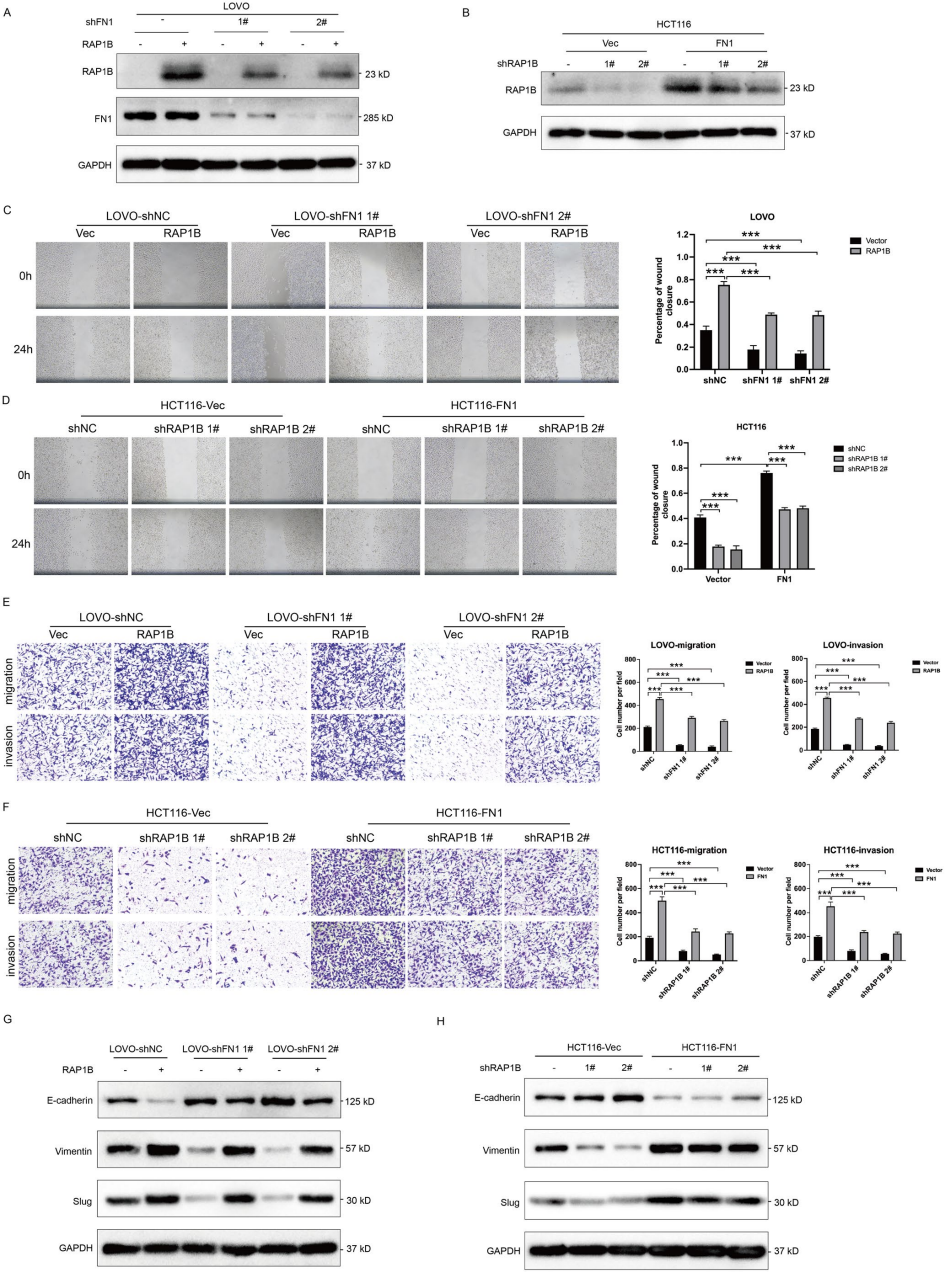
FN1 promotes cell migration, invasion, and EMT via RAP1B. (A) Western blot analysis of RAP1B in HCT116‐vector/HCT116‐FN1 cells transfected with RAP1B shNC or sh RAP1B. (B) Western blot analysis of RAP1B in LOVO‐shNC/LOVO‐shFN1#1/LOVO‐shFN1#2 transfected with vector or RAP1B‐overexpressing plasmid. (C) Wound healing analysis of HCT116‐vector/HCT116‐FN1 cells transfected with RAP1B shNC or shRAP1B. (D) Wound healing analysis of LOVO‐shNC/LOVO‐shFN1#1/LOVO‐shFN1#2 transfected with vector or RAP1B‐overexpressing plasmid. (E, F) Transwell and transwell‐matrigel assays were performed to assess migration and invasion ability of FN1‐knockdown and FN1‐overexpression cell lines. Representative images (left panel) and quantification (right panel) are shown as indicated. (G, H) Western blot analysis of EMT‐related proteins for LOVO‐shNC/LOVO‐shFN1#1/LOVO‐shFN1#2 and HCT116‐vector/HCT116‐FN1. ***p < 0.001.

To further investigate whether FN1 regulates colon cancer migration and metastasis in vitro through RAP1B, we introduced RAP1B shRNAs in FN1‐overexpression HCT116 cells, and transfected FN1‐knockdown LOVO cells with RAP1B overexpression plasmid. The expression of RAP1B was confirmed by western blot (Figure 5A,B). Wound healing assay showed that RAP1B knockdown in FN1‐overexpression HCT116 cells partially blocked the increased cell healing ability induced by FN1 overexpression. RAP1B overexpression in FN1‐knockdown LOVO cells rescued the decreased cell healing ability caused by FN1 knockdown (Figure 5C,D). Consistently, transwell assays showed that RAP1B downregulation in FN1‐upregulation HCT116 cells partially inhibited the increased cell migration and invasion abilities by FN1 upregulation alone, while RAP1B upregulation in FN1‐downregulation LOVO cells restored the decreased cell migration and invasion abilities by FN1 downregulation alone (Figure 5E,F). EMT plays a vital role in endowing tumour cells with the ability to migrate, invade, and metastasize. We also assessed whether FN1 mediated colon cancer EMT via RAP1B. The EMT‐related markers were detected. The decreased expression of E‐cadherin and increased Vimentin and Slug expression in FN1‐overexpression HCT116 cells were partially blocked by RAP1B knockdown (Figure 5G). The elevated level of E‐cadherin and reduced levels of Vimentin and Slug in FN1‐knockdown LOVO cells were rescued by RAP1B overexpression (Figure 5H). These data suggest that FN1 promotes colon cancer cell migration, invasion, and EMT via RAP1B. RAP1B is the effective target of FN1 in colon cancer.

### 3.6 FN1‐RAP1B Promotes the Binding of CREB to FN1 and TIMP1 Promoter via Akt/CREB Signalling

**FIGURE 6:**
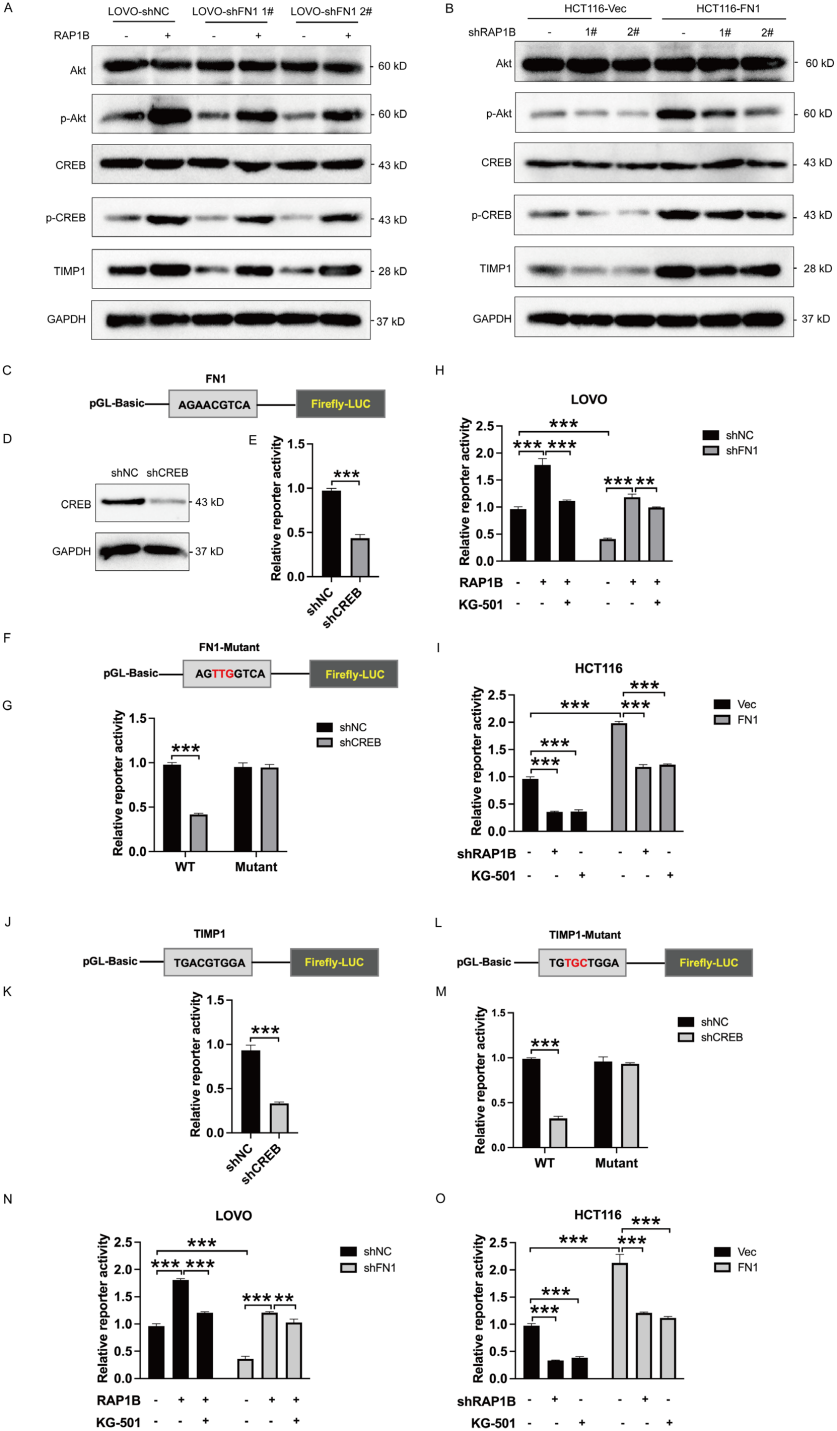
FN1‐RAP1B promotes the binding of CREB to FN1 and TIMP1 promoter via Akt/CREB signalling. (A) The protein level of Akt, p‐Akt, CREB, p‐CREB, TIMP1 was detected in HCT116‐vector/HCT116‐FN1 transfected with RAP1B shNC or shRAP1B. (B) The protein level of Akt, p‐Akt, CREB, p‐CREB, TIMP1 was detected in LOVO‐shNC/LOVO‐shFN1#1/LOVO‐shFN1#2 transfected with vector or RAP1B‐overexpressing plasmid. (C) Schematic diagram of the FN1‐promoter luciferase reporter plasmid. (D) Western blot of CREB in LOVO cells transfected with shCREB or shNC. (E) The luciferase reporter assay containing the region of the FN1 promoter was applied in LOVO cells transfected with shCREB or shNC. (F) The schematic diagrams of the wild‐type FN1 promoter with putative CREB binding site and mutated FN1 promoter. The wild‐type and mutated sites were indicated in grey box. (G) The luciferase reporter assay containing the wildtype and mutant region of the FN1 promoter was performed in LOVO cells transfected with shCREB. (H) The luciferase reporter assay was applied in HCT116‐vector and HCT116‐FN1 cells transfected with RAP1B siRNA with KG‐501 treatment. (I) The luciferase reporter assay was performed in LOVO‐shNC and LOVO‐shFN1 cells transfected with the RAP1B‐overexpressing plasmid with KG‐501 treatment. (J) The schematic diagram of wild‐tpye TIMP1 promoter with putative CREB binding site. (K) The luciferase reporter assay containing the region of the TIMP1 promoter was applied in LOVO cells transfected with shCREB or shNC. (L) The schematic diagrams of the wild‐type TIMP1 promoter with putative CREB binding site and mutated TIMP1 promoter. (M) The luciferase reporter assay was applied in HCT116‐vector and HCT116‐FN1 cells transfected with RAP1B siRNA with KG‐501 treatment. (N) The luciferase reporter assay was performed in LOVO‐shNC and LOVO‐shFN1 cells transfected with the RAP1B‐overexpressing plasmid with KG‐501 treatment. (O) The luciferase reporter assay was performed in HCT116‐vector and HCT116‐FN1 cells transfected with shRAP1B with KG‐501. ***p < 0.001.

To further deeply investigate the detailed mechanism by which FN1 regulates RAP1B in colon cancer, we detected the expression of Akt/CREB signalling pathway. FN1 overexpression increased the expression of TIMP1, the phosphorylation level of Akt and CREB, which was partially blocked by RAP1B knockdown (Figure 6A). At the same time, knockdown of FN1 (shFN1 #2 was used in the later study for its better knockdown efficiency) decreased the expression of TIMP1, the phosphorylation level of Akt and CREB, which was partially rescued by RAP1B overexpression (Figure 6B). It has been reported that the transcription factor CREB1 could regulate FN1 expression by activating FN1 mRNA transcription [26]. Therefore, we hypothesized that FN1‐RAP1B mediated phosphorylated CREB might transcriptionally regulate FN1 expression. Thus, we constructed the FN1‐promoter luciferase reporter plasmid, which was used to evaluate CREB‐dependent FN1 signalling regulation in HCT116 and LOVO cells (Figure 6C). It was observed that CREB knockdown reduced the transcription activity of FN1 (Figure 6D,E). In addition, we also constructed mutant FN1 luciferase reporter plasmid, which could not be regulated by CREB (Figure 6F). Results showed that knockdown of CREB did not alter the transcription activity of FN1 (Figure 6G). These results indicate that CREB could promote FN1 transcription activity. Furthermore, we evaluated the regulation of FN1‐RAP1B mediated‐CREB on FN1 transcription activity by using the CREB inhibitor KG‐501. The increased FN1 transcription activity induced by RAP1B upregulation was found to be partially suppressed with treatment of KG‐501. The decreased FN1 transcription activity caused by FN1 knockdown could be rescued by RAP1B upregulation, which was blocked with treatment of KG‐501 (Figure 6H). Subsequently, FN1 overexpression could increase FN1 transcription activity, which was partially blocked by RAP1B knockdown. And, the above increased phenotype could also be blocked by KG‐501 treatment (Figure 6I). Taken together, these data suggest that FN1 upregulation promotes CREB phosphorylation and activate CREB by regulating RAP1B, resulting in the binding between CREB and FN1 promoter region to exert transcription activity on FN1, thus upregulating the expression of FN1.

Further, the above findings indicate that FN1 overexpression also promotes TIMP1 expression via RAP1B. Since there was a binding motif of CREB and the TIMP1 promoter region, we also constructed a TIMP1 luciferase reporter plasmid (Figure 6J). As expected, CREB knockdown could also suppress the transcription activity of TIMP1 (Figure 6K). Moreover, CREB knockdown could not change the mutant TIMP1 luciferase activity (Figure 6L,M). These data suggest that CREB also regulates TIMP1 transcription activity, thus elevating TIMP1 expression. Next, we also investigated the regulation of FN1‐RAP1B‐CREB on TIMP1 transcription activity. The decreased TIMP1 transcription activity induced by FN1 downregulation was rescued by RAP1B overexpression, which was suppressed by co‐treatment with KG‐501 (Figure 6N). The increased TIMP1 luciferase activity caused by FN1 overexpression was blocked by RAP1B knockdown or KG‐501 treatment (Figure 6O). In sum, these results indicate that CREB was also a transcription factor of TIMP1, which was regulated by FN1‐RAP1B.

### 3.7 FN1‐RAP1B Promotes Distant Metastasis In Vivo

**FIGURE 7:**
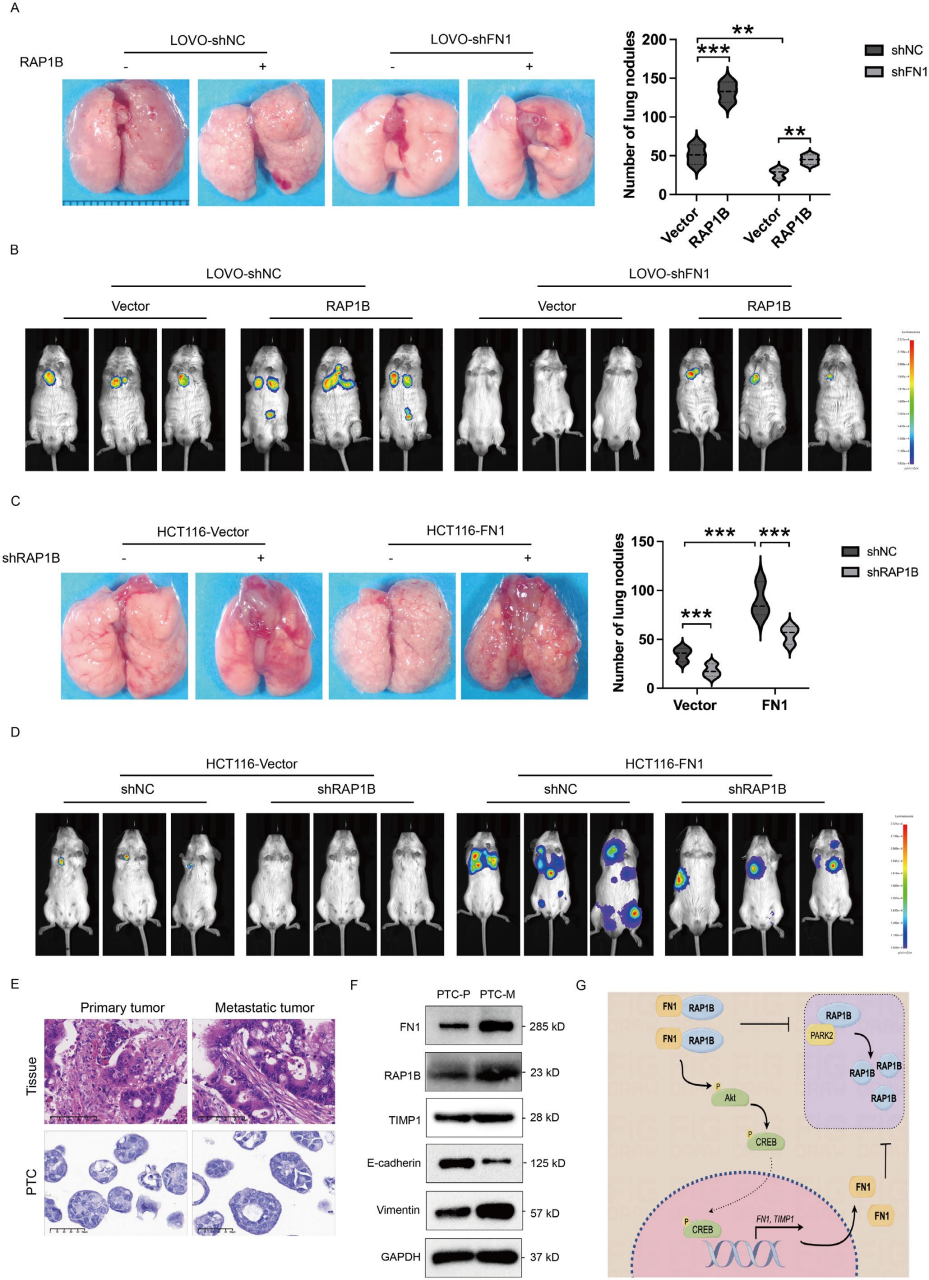
FN1‐RAP1B promotes distant metastasis in vivo. (A) Representative images of lung metastasis nodules from indicated group of mice. Quantitative analysis of lung metastasis nodules from corresponding mice (right). (B) Representative bioluminescent images of colon cancer in NGS mice with tail vein injection of LOVO‐shNC and LOVO‐shFN1 transfected with or without shRAP1B. (C) Representative images of lung metastasis nodules from the indicated group of mice. Quantitative analysis of lung metastasis nodules from corresponding mice (right). (D) Representative bioluminescent images of colon cancer in NGS mice with tail vein injection of HCT116‐vector and HCT116‐FN1 with or without shRAP1B. (E) Representative images of H&E staining of tumour tissues and PTCs from colon cancer samples. (F) Western blot analysis of FN1, RAP1B, TIMP1, E‐cadherin and Vimentin in primary PTCs and metastasis PTCs. (G) Schematic representation of the FN1‐RAP1B/Akt‐CREB positive feedback loop promoting colon cancer metastasis. **p < 0.01, ***p < 0.001.

To further validate the role of the FN1‐RAP1B axis in colon cancer metastasis in vivo, LOVO‐shFN1 cells and/or LOVO‐RAP1B‐overexpressing cells were injected into the lateral tail veins of the nude mice. The lung metastasis nodules displayed the most in the RAP1B‐overexpressing group and the least in the FN1‐silence group, and were partially restored by both the FN1‐silence and RAP1B‐overexpressing groups (Figure 7A). Besides, the distribution and accumulation of colon cancer metastasis were also visualised by the imaging system (Figure 7B). The pulmonary metastases were distributed widely in the mice injected with LOVO‐RAP1B‐overexpressing cells, while this phenotype was partially blocked by both FN1‐silence and RAP1B‐overexpressing (Figure 7B). On the contrary, mice injected with HCT116‐FN1‐overexpressing cells showed a huge amount of white clump‐like dense tissues on the lungs, which was partially blocked by RAP1B‐knockdown, and no obvious clumps were observed on the lungs of the LOVO‐RAP1B‐silence group (Figure 7C). Consistently, the number of lung metastasis tumours was significantly elevated in the FN1‐overexpressing group compared with the control group, which was partially restored by the FN1‐overexpressing and RAP1B‐silenced groups (Figure 7D). Taken together, this data demonstrates that FN1 can promote colon cancer metastasis in vivo via RAP1B.

Finally, we also detected the expression of FN1, TIMP1, and RAP1B in ex vivo by establishing the patient‐derived tumour‐like cell clusters (PTCs) from primary and metastatic colon cancer tumour tissues. The Haematoxylin and eosin staining analysis showed that there were high morphological similarities between colon cancer tissues and PTCs (Figure 7E). The mRNA and protein levels of FN1 and RAP1B were positively and highly expressed in metastatic PTCs compared to primary PTCs (Figure 7F), along with the high expression of Vimentin and low expression of E‐cadherin. To sum up, these findings suggest that FN1 can enhance colon cancer metastasis in vivo through RAP1B.

## 4 Discussion

In the present study, we demonstrated that FN1 was highly expressed in the primary and metastatic colon cancer and positively related to the poor prognosis of colon cancer. Overexpression of FN1 promoted gastric cancer migration, invasion, EMT, and metastasis via RAP1B in vitro and in vivo. Mechanistically, FN1 relieved RAP1B ubiquitination by suppressing its binding to the ubiquitination ligase PARK2, improving RAP1B protein stability and activating the Akt signalling pathway, resulting in the phosphorylation and activation of the transcription factor CREB. Interestingly, CREB was also involved in the induction of FN1 and TIMP1 expression, thereby leading to a positive feedback loop in colon cancer metastasis (Figure 7G). Moreover, FN1 and RAP1B were also positively upregulated in PTCs from primary and metastatic colon cancer tumour tissues.

Increasing studies have reported that FN1 plays vital roles in the metastasis of multiple tumours. It was found that increased expression of FN1 mediated by NK cell receptor NKp46 could alter primary tumour architecture and result in the decreased metastases formation [27]. FN1 and TGFβ was discovered to constitute positive reciprocal regulation loop in the prevention of cisplatin together with paclitaxel on breast cancer metastasis [28]. A recent study reported that FN1 mRNA 3′‐UTR could facilitate the invasion and metastasis of gastric cancer [29]. Another report showed that PTAL‐miR‐101‐FN1 Axis could promote EMT and invasion‐metastasis in serous ovarian cancer [30]. A previous study found that FN1 was involved in IRE1α regulated metastatic potential of colon cancer cells [16]. Although some studies have shown that FN1 modulates several cancers malignant progression including colon cancer, the specific mechanism of FN1 regulating the migration, invasion, EMT and metastasis of colon cancer remains poorly understood.

In this work, we performed RNA‐seq and observed that FN1 and TIMP1 were the most upregulated genes between metastasis and primary colon cancer tissues as well as between primary colon cancer tissues and adjacent normal tissues. Furthermore, we validated the expression and prognostic value of FN1 in GEO datasets and tumour samples. Then we identified that FN1 promoted colon cancer migration, invasion and EMT in vitro.

Next, we predicted that RAP1B was the interacting protein of FN1. KEGG enrichment showed that FN1 related genes were mainly enriched in RAP1 and RAS signalling pathway, in which Ras‐related protein RAP1B was involved and played key roles in cancer metastasis. The interaction between FN1 and RAP1B was predicted by bio‐informatics analysis and then verified through the endogenous and exogenous immunoprecipitation. The protein level of RAP1B but not mRNA level was found to be changed when FN1 was overexpression or knockdown, suggesting that FN1 may modulate the protein stability of RAP1B through post‐translational modification. Previous research has reported that RAP1B could be regulated by the ubiquitin‐proteasome pathway, playing key roles in the control of cell polarity, migration, and cell transformation [31]. Thus, we treated cells with the protein synthesis inhibitor CHX at different time. The protein degradation of RAP1B was significantly prevented in FN1‐overexpressing cells. Treatment of MG132, RAP1B protein degradation slowed down in FN1‐knockdown cells. These results indicated that FN1 affects RAP1B protein stability via the ubiquitin proteasomal degradation. Furthermore, the ubiquitination modification of RAP1B was significantly reduced in MG132‐treated FN1‐overexpressing cells compared to vector control. Taken together, we demonstrated that FN1 regulates RAP1B protein stability via the ubiquitin‐proteasome system.

Based on the bioinformatics analysis of our RNA‐seq data and the GEO dataset, we identified that highly expressed RAP1B enriched the ubiquitination pathway in primary and metastasis colon cancer compared to normal tissues. The ubiquitin ligase PARK2 was predicted to interact with RAP1B via the online database. The binding of RAP1B and PARK2 was confirmed by Co‐IP experiments. Moreover, the interaction between RAP1B and PARK2 was decreased with the overexpression of FN1. The ubiquitination modification of RAP1B enhanced by PARK2 overexpression was decreased by FN1 upregulation. These data suggest that FN1 inhibits the ubiquitination of RAP1B though relieving RAP1B binding to PARK2.

PARK2 (also named Parkin) has been shown to function as an E3 ubiquitin ligase and controls several cellular processes [32, 33], which has been reported as a tumour suppressor gene in various cancers. It was reported that PARK2 associated with YAP protein and promoted YAP ubiquitination via the proteasome‐dependent degradation to regulate oesophageal squamous carcinoma progression [34]. Another study in breast cancer showed that PARK2 promotes mitochondrial pathway of apoptosis and antimicrotubule drugs chemosensitivity via degradation of phospho‐BCL‐2 [35]. It was found the PARK2 knockout mice were susceptible to colon cancer, suggesting that PARK2 acted as an important contributor for oncogenic process [36]. A previous study revealed that the deletion of PARK2 occurs frequently in sporadic colorectal cancer and accelerates the adenoma development in Apc mutant mice [37]. However, the role of PARK2 in colon cancer metastasis has never been reported. It is the first study that clarify that FN1 could suppress the binding of RAP1B to PARK2 to stabilise RAP1B protein, thereby activating Akt signalling pathway and promoting gastric cancer migration and metastasis.

Accumulating studies suggest that the Akt signalling pathway plays important roles in cancer progression and metastasis [38, 39, 40, 41]. For example, RNF12 is regulated by AKT phosphorylation and promotes TGF‐β driven breast cancer metastasis [41]. Exosomal circTUBGCP4 promotes vascular endothelial cell tipping and colorectal cancer metastasis by activating the Akt signalling pathway [42]. Previous studies report that CREB signalling is associated with cancer metastasis [43, 44, 45, 46]. IGF1 induced SOX12 expression via the PI3K/AKT/CREB pathway, forming an IGF1/CREB/SOX12 feedback loop that contributed to gastric cancer metastasis [47]. CT45A1 overexpression in breast cancer cells promoted epithelial‐mesenchymal transition, migration, and invasion through activating the ERK and CREB signalling pathways [48]. In this study, the highly expressed FN1 phosphorylated and activated Akt and CREB, thus promoting the nuclear translocation of CREB and increasing FN1 and TIMP1 promoter activity, leading to the upregulation of the expression of FN1 and TIMP1. Therefore, the positive regulatory feedback loop was established, which contributed to the migration, invasion, and metastasis of colon cancer.

There were some limitations in this study. Firstly, the mechanism of FN1 promoting colon cancer metastasis was investigated mainly in cell lines, animal models, and several clinical samples, lacking a large scale of clinical research validation. In addition, this research only suggested that the FN1/RAP1B/CREB loop may be a therapeutic target, but did not further explore specific treatment strategies and drug development for this target. Future research will continue to address these issues.

In conclusion, we demonstrate that highly expressed FN1 is associate with colon cancer metastasis and poor prognosis. FN1 promotes colon cancer migration, invasion, EMT and metastasis though RAP1B. FN1 enhances the protein stability of RAP1B though inhibiting its binding to PARK2, thus activating the Akt/CREB signalling pathway. The activated CREB can also directly bind to the promoter of FN1 and TIMP1 and enhance FN1 and TIMP1 expression. Our study provides strong support that the vital role of FN1/RAP1B/CREB loop during colon cancer metastasis. Taken together, our results reveal that targeting FN1/RAP1B/CREB‐mediated tumour interactions might be an attractive therapeutic strategy for metastasis colon cancer patients.

## Author Contributions

**Zhonghe Ji:** conceptualization (equal), project administration (equal), supervision (equal), writing – original draft (equal). **Xinbao Li:** data curation (equal), formal analysis (equal), investigation (equal), validation (equal), writing – original draft (supporting). **Yadong Wang:** data curation (equal), formal analysis (equal), investigation (equal), validation (equal), writing – original draft (supporting). **Xinjing Zhang:** data curation (equal), formal analysis (equal), investigation (equal), validation (supporting). **Zhiran Yang:** data curation (equal), formal analysis (equal), investigation (supporting), validation (equal). **Yanbin Zhang:** data curation (equal), formal analysis (equal), investigation (equal), validation (supporting). **Junhui Yu:** data curation (supporting), formal analysis (supporting), investigation (supporting), validation (supporting). **Chao Gao:** investigation (supporting), methodology (equal), resources (equal). **Guojun Yan:** investigation (equal), methodology (supporting), resources (equal). **Lijun Yan:** investigation (supporting), methodology (supporting), resources (supporting). **Kai Zhang:** investigation (supporting), methodology (supporting), resources (supporting). **Jinghan Pan:** investigation (supporting), methodology (supporting), resources (supporting). **Songlin An:** conceptualization (equal), supervision (equal), writing – review and editing (equal).

## Ethics Statement

This study involving human participants was approved by Ethics Committees of Beijing Shijitan Hospital (sjtky11‐1x‐2021(12)).

## Conflicts of Interest

The authors declare no conflicts of interest.

## Supporting information


**Table S1.** GSEA analysis of RAP1B related genes.

## Data Availability

The data are available from the corresponding author for reasonable requests.
